# Seeing Beyond Salience and Guidance: The Role of Bias and Decision in Visual Search

**DOI:** 10.3390/vision3030046

**Published:** 2019-09-11

**Authors:** Alasdair D. F. Clarke, Anna Nowakowska, Amelia R. Hunt

**Affiliations:** 1 Department of Psychology, University of Essex, Colchester CO4 3SQ, UK; 2 School of Psychology, University of Aberdeen, Aberdeen AB24 3FX, UK

**Keywords:** visual search, eye movements, attention, strategy, decision

## Abstract

Visual search is a popular tool for studying a range of questions about perception and attention, thanks to the ease with which the basic paradigm can be controlled and manipulated. While often thought of as a sub-field of vision science, search tasks are significantly more complex than most other perceptual tasks, with strategy and decision playing an essential, but neglected, role. In this review, we briefly describe some of the important theoretical advances about perception and attention that have been gained from studying visual search within the signal detection and guided search frameworks. Under most circumstances, search also involves executing a series of eye movements. We argue that understanding the contribution of biases, routines and strategies to visual search performance over multiple fixations will lead to new insights about these decision-related processes and how they interact with perception and attention. We also highlight the neglected potential for variability, both within and between searchers, to contribute to our understanding of visual search. The exciting challenge will be to account for variations in search performance caused by these numerous factors and their interactions. We conclude the review with some recommendations for ways future research can tackle these challenges to move the field forward.

Searching is a familiar, sometimes frustrating, part of daily life. We search our homes and offices for keys, glasses, wallets and bags. We look for street signs, cars in parking lots, items in the supermarket, children on a playground and friends in crowded restaurants. With all this searching, one might expect us to be experts, but searching is often effortful, time-consuming and error-prone. In the laboratory, even highly-simplified versions of search tasks illustrate why this is the case: unlike many other tasks used in vision and attention research, visual search is a complex task that involves visual processing in both central and peripheral vision, controlling our attention to filter out distracting visual features, coordination of vision and attention with eye (and sometimes head) movements and a dependent sequence of one or more decisions about when and where to move attention and the eyes to sample information. Finally, visual search involves high-level strategic decisions about how to search and for how long to search without finding anything before giving up. Seen in this light, it is not surprising that the performance of visual search is often slow and highly variable.

Despite its complexity, visual search is a realistic task that is easy to control and manipulate in experimental settings, and it is a popular tool for studying a range of questions about perception and attention. Less commonly, visual search can also be used to study strategy and decision-making. Previous studies of visual search can be loosely categorised by whether observers make a single fixation or if search unfolds over multiple fixations. Single-fixation search makes heavy use of psychophysical methods (i.e., [1,2]). Theories in this literature have established how representations of the stimulus interact with the limits of attention to inform decisions as to the presence or absence of the target. However, decision here is in the context of psychophysical signal detection theories. In our literature review below, we focus on multiple-fixation search (also sometimes referred to as “foraging”, although this label is usually reserved for multiple-target search), and when we talk of decisions, we mean in the context of the wider field of cognitive psychology, that is, how we select between different courses of action. While Palmer et al. [1] (p. 1256), for example, discussed how their psychophysical theory of visual search could be extended to multiple-fixations, in terms of search strategy, they went no further than suggesting “The choice of the location of the next fixation is probably a function of the information in the periphery and the observer’s strategy.” In what follows, we hope to convince the reader that this second component, the observer’s strategy, is highly idiosyncratic, accounts for a large proportion of variation in the speed and success of search and is heavily influenced by both oculomotor and decision biases.

In this review, we will first briefly describe some of the important theoretical advances about perception and attention that have been gained from studying visual search. Our understanding of how visual features and selective attention operate and interact during a single fixation of visual search is well developed, thanks to several decades of research. We also have an emerging understanding of the influence of higher level factors like reward and serial dependences on these within-fixation processes. However, search performance also depends on decisions about where to fixate, and over a series of these fixation decisions, the effect on performance accumulates rapidly. We will argue that a focus on understanding the contribution of biases, routines and strategies to visual search performance will help complete our understanding and lead to new insights, not only about how we search, but about how these decision-related processes interact with perception and attention more generally.

## 1. Perception and Attention in Visual Search: A Brief Summary

How perceptual processes and attention interact to help us define and find visual targets is the subject of a large body of research. Many excellent reviews have recently been published on this topic. For example, Volume 29 of *Current Opinion in Psychology* contains several papers that give up-to-date reviews and opinions on perception and attention in visual search [3,4,5,6,7,8]. We direct the reader to these papers for a more thorough and critical treatment of the themes covered in this section. In what follows, we briefly summarize three key themes of this literature as the background for the main topic of this review, which is the decision processes involved in search over multiple fixations.

### 1.1. Visual Salience

The influence of low-level visual information on search is typically taken as synonymous with the influence of visual salience. The first formal computational model for visual salience was developed over 20 years ago [9], and early work used visual search paradigms to explore the model’s behaviour [10]. Since then, visual salience has developed into its own sub-field within vision science, focused less on explaining human performance in visual search tasks and more on accounting for, and predicting, the patterns of fixations that human observers make when viewing complex (usually photographic) stimuli. In many respects, this body of work is one of the success stories for psychology in the early 21st Century, with the current state-of-the-art models (based on machine learning and convolutional neural networks) approaching ceiling performance [11,12]. While these models offer impressive performance in predicting which regions of an image are likely to be visually inspected, the move to a computer vision approach focused on performance metrics has led to questions about whether these models are actually modelling visual salience or acting more as a weighted object detector [13,14].

Prior to these models, debate over the relative importance of low-level and mid-to-high-level information in guiding fixations and attention was common (e.g., [15,16]). However, the success of these models suggests that drawing distinctions between these levels of analysis may not be necessary. The processing of low and high level features in the brain is highly interconnected; we should question whether it is appropriate to model them as distinct sets [17].

### 1.2. Attentional Guidance and Control Settings

The literature on visual search over the past 30 years has been dominated by the guided search model [18], which is now in its fifth iteration [19]; the full version of the fourth iteration can be found here [20]. Many ideas in the guided search model evolved from Treisman’s Feature Integration Theory of attention (FIT) [21], in which a small set of basic visual features, such as colour and orientation, are pre-attentive, that is, they are processed in the absence of attention. Conjoining these basic features, FIT proposes, is an operation that depends on attention. Targets that can be defined based on pre-attentive features will “pop out” and be found quickly, while a target defined by conjunctions of features will require directing spatial attention to potential targets until it is found, a much slower and more demanding process. The time to find a conjunction target increases in proportion to the number of items in the set. This per-item increase in reaction time with search set size, known as the search slope, is taken as an index of the attentional demands of finding the target. Guided search builds on FIT by modelling search as a process of elimination, whereby the target will have “guiding features”, which can be used to limit attention to the subset of items in the scene that share this feature. For example, if your own car is red and you cannot remember where you parked it, you only need to check the other red cars in the parking lot. In guided search, a pre-attentive visual feature like colour can be identified and used by attention rapidly and in parallel across the visual field. Once you have narrowed down the set, a more labourious process of serial inspection begins. There is broad agreement about the general premise of the guided search model, although some of the details are the subject of lively debate.

The flip side of guidance is distraction. Some types of distractors can impede search even when they do not share obvious features with the target. For example, sudden onsets have consistently been shown to capture attention “reflexively”, that is irrespective of how the target of search has been defined [22], and the presence of a sudden onset will slow reaction times to find a target. The existence of attentional capture has led to the suggestion that some biologically-relevant or visually-distinctive information might act as a kind of “circuit-breaker” on attentional guidance, attracting attention even when it is not relevant to the current task [23]. The list of visual features that produce truly reflexive capture is shorter than the list of features capable of guiding attention, and sudden onsets may be the only feature that stands uncontended. In most other circumstances, whether and which distractors capture attention can be argued to depend largely on what features are being used to guide attention [24,25] or how much attention is engaged in the task [26]. The setting or tuning of attention to detect particular features is commonly known as attentional control settings [27].

In both guidance and distraction, debate persists around many categories of stimuli that might be more or less easy to ignore or find, depending on their status in the visual system or presumed biological relevance [7,28,29]. These special features are often referred to as “pre-attentive”, meaning they do not require attention to be detected and used to control behaviour [21]. They are also often referred to as “bottom-up”, suggesting they control attention in a feed-forward (rather than re-entrant) manner (e.g., [30]). These interpretations suggest the list of pre-attentive or bottom-up features should be invariant, that is, while there may be some disagreement around how exactly to categorize a feature, applying the same criteria should lead to the same list across individuals and over time. A serious issue with this, however, is recent questions around how predictable and invariant the guiding properties of a visual feature really are (e.g., [31]). The ease with which certain features can be used to guide attention is determined in part by how frequently, or recently, we have used those features to find a target (e.g., [6]), implying these could vary over time. Objects that have been previously associated with rewards can also capture attention later, when they are no longer relevant [32]. Thus, the historical distinction between “bottom-up” and “top-down” control of attention has begun falling out of favour, for similar reasons as for the distinction between the relative contribution of “low-level” and “high-level” information in determining fixations. As we have come to understand the details of how attention is guided by information, these categories have become increasingly blurred.

### 1.3. Eye Movements in Visual Search

While the above body of work has been very useful in helping us uncover how our attentional systems deal with different types of information (features such as colour, orientation and shape), it has either overlooked eye movements in visual search or treated them as synonymous with attention. Hulleman and Olivers [33] argued that progress in the field has been hampered by this approach, which tends to treat individual search items as the unit of analysis and neglects the varying effect of peripheral vision on different types of features. They proposed replacing search items with fixations and a functional viewing field as the base unit of analysis in visual search experiments. One immediate advantage of such a shift in conceptual unit is that it allows theories of visual search to go beyond describing visual search through simple arrays of clearly-defined items to tasks such as searching X-ray scans, textures and photographs of natural scenes. An earlier example of this approach was the target acquisition model [34], a computational model based on a pyramid of filters that are used to generate an activation map that represents the similarity between points in the search image (after transforming to take the retina into account) and the target. The model’s search strategy is to simply move the eyes to the centre of mass of the activation map. While this allows it to generate centre-of-gravity fixations that are often seen in human scan-paths (i.e., [35]), there is no notion of varying search strategies, and while the problem of speed/accuracy trade-offs with relation to absent target responses was discussed, this decision process was not implemented. It should also be noted that eye movements do not always help search (e.g., [36,37,38]). However, given the nature of our foveated visual system, accounting for eye movements is clearly an important part of any theory of visual search.

## 2. Biases and Strategy

Searchers typically have the freedom to move (or not) their eyes and bodies around during natural search. It is important to understand the contribution of all the factors described in the previous section—low and high-level visual information and how attention is guided and distracted by these properties—to the selection of fixations during search. However, there is another layer of search behaviour that is not captured by these factors. This is the layer that involves biases, decisions, and heuristics that influence how we position our eyes. This is the primary focus of the rest of our review. As we will demonstrate, fixation strategies have a large impact on our search speed and success and vary dramatically from one individual to another.

### 2.1. Optimality vs. Stochasticity

Given the amount of time we spend looking for things in our day-to-day lives, one might expect us to have evolved and/or developed efficient search strategies. Consistent with this possibility, Najemnik and Geisler developed an influential model of visual search based on an ideal search strategy, and the model matched the number of fixations it takes humans to find a target [39,40]. The model directs each fixation during search to the location that will provide the most information about the target’s location, taking into account which locations have already been fixated and the difficulty of spotting the target at different eccentricities. As such, it finds a target in the smallest possible number of fixations. That humans can match this level of efficiency suggests that we may be approaching performance that is as good as it gets, given the limitations of our visual systems. This approach has been expanded upon by Hopper and Rothkopf [41] who argued that humans are capable of constructing multi-step plans for eye movements in visual search tasks that maximize information gain over more than just the next fixation.

**Figure 1:**
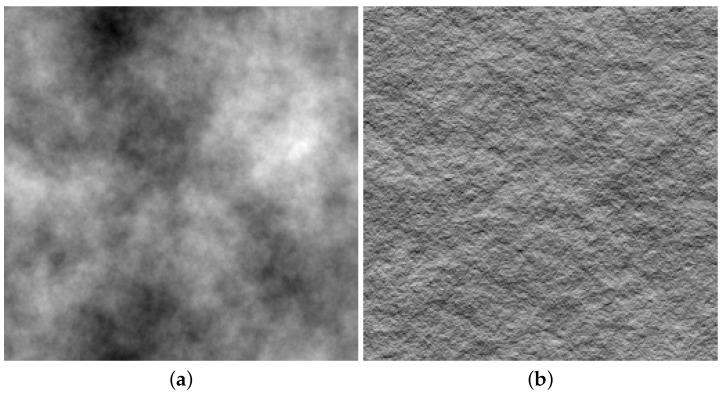
Example stimuli from [42]. (**a**) shows a two-dimensional 1/f-noise stimulus, similar to those used by [40]. (**b**) shows the effect of treating this 1/f stimulus as a surface texture and rendering with illumination from above.

There is an important caveat to note. Najemnik and Geisler [40] acknowledged that they did not consider their work a plausible model for human search mechanisms, but instead, the human visual system makes use of heuristics that produce some of the rational behaviour exhibited by their model. The chief reason for this is the extremely high computational load associated with calculating expected information gain for every possible fixation position ahead of each eye movement. For the artificial search scenes of 1/f noise used by Najemnik and Geisler [40] (see Figure 1), with uniform and easily-estimated levels of target visibility, the calculation of an optimal fixation strategy is tractable, but resource-intensive. Considering we move our eyes 2–3 times each second during search and estimating target visibility is not often so straightforward as this, it seems likely that we take some short cuts.

The idea of an “ideal” searcher also stands in contrast to our modelling work on visual search behaviour in 1/f textures [42] (see Figure 1) in which visual search is thought of as a random walk of saccades (see also [43]). After each saccade is made, there is a probability that the target will be detected, which is modulated by how far the target is from the current fixation location and the contrast of the texture. These detection probabilities are fitted to empirical psychophysical data, and importantly, while saccades are selected randomly, we sampled from the population of saccades participants made from the same region of the search array. Unlike the optimal model, this stochastic selection process has no information about the display or knowledge of what has already been fixated. Nonetheless, the stochastic search model, just like the optimal search model, takes a similar number of fixations to find the target as human searchers do. This suggests that the stochastic model also adequately describes human search, while being computationally far simpler than an ideal searcher.

### 2.2. Oculomotor Biases

It has been increasingly apparent that oculomotor biases have a large role to play in explaining the patterns of fixations made during scene viewing and visual search. At the very least, these should be considered a significant contribution to variance that needs to be taken into consideration when developing models of eye movement behaviour. However, a stronger statement is that these biases serve a functional role and can be thought of as evolved heuristics that allow observers to search their environment efficiently without needing to carry out the complex calculations necessary to implement the optimal strategy. The utility of these heuristics could explain why an optimal [40] and a stochastic [42] search model can describe human search behaviour equally well.

Perhaps the simplest demonstration of this point is the central bias [44,45]. Observers were tasked with searching photographs of everyday scenes for an embedded Gaussian luminance target. Recordings of eye movements made during search showed that observers preferentially fixate on the central region of the stimuli, even for photographs in which the interesting (in terms of salience and semantic content) information is located away from the centre. Furthermore, it has been demonstrated (e.g., [46]) that adding a central bias to salience models significantly improves their ability to predict which regions of an image will be fixated.

We also see biases in saccadic direction and amplitude. The distribution of directions shows a pronounced preference for saccades to the left and right (i.e., [47]). Human observers also exhibit a strong bias in the magnitude of the saccades they make, with distributions often having a lower mean and larger positive skew than what can be accounted for by a naive salience model [48]. A smaller, but robust, bias is the preference of observers to direct their attention initially to the left half of a scene [49,50,51]. Another behaviour that can be thought of as a heuristic and strategy is coarse-to-fine search [52]. When faced with a new search stimulus, observers start by making long saccades and short fixations. As the search progresses, saccadic amplitude decrease while the length of fixation duration increases. Interestingly, there was little influence of target salience on this behaviour, leading Over et al. [52] to suggest that an intrinsic coarse-to-fine heuristic for visual search is used, even when such a strategy is not optimal. Inhibition of return [53] and saccadic momentum (e.g., [54]) have been proposed to bias eye movements to new locations and avoid “wasting” eye movements by revisiting locations that have recently been fixated, though with different underlying mechanisms, time courses, and effects [55].

Following this line of thought, we have built on the stochastic search model discussed above [42] and developed a “blind” model of eye movements during scene viewing [56]. This model does not take any inputs and simply models the probability of making a saccade to $\left(x_{i},y_{i}\right)$ given a fixation at $\left(x_{i-1},y_{i-1}\right)$. These probabilities are estimated as a truncated multivariate Gaussian distribution, fitted over a range of datasets, and the model is intended to act as a strong baseline to which more sophisticated, image-processing models can be compared. Alternatively, it can be thought of as a computational model of some of the oculomotor biases outlined above.

### 2.3. Decision Biases

The division between eye movements guided by information gain on the one hand [40] and guided by heuristics and biases on the other [42] is reminiscent of a classic distinction in the animal cognition literature between actions and habits, where actions are responses guided by knowledge of the consequences and habits are repetitions of behaviours that have previously been reinforced [57]. We have been specifically considering visual search in this review and considering the process of selecting fixations as a way of measuring strategies and biases in how information is sampled during speeded search, as well as during visual inspection more generally. However, it is important to consider whether the principles and biases guiding fixation selection are specific to the visual and oculomotor systems, versus a reflection of principles and biases guiding human choices more generally. There are many reasons to expect the decision processes guiding eye movements to be unique to this system: these decisions are made with a very high frequency (2–3 times per second during visual search) and require very little effort to execute, relative to other decisions. Based on these properties, one might expect eye movements to be particularly “thoughtless”, perhaps even meeting Dickinson’s definition of a habit, and more likely to be guided by heuristics than by careful consideration of various options and their consequences.

A clever demonstration of “thoughtless” eye movement heuristics was devised by Morvan and Maloney [58]. In their task, there were two boxes in which a target (a small dot) could appear, presented at varying distances apart. A central box was always presented at the centre, but never contained the target. The participant moved their eyes to one of the boxes, and as soon the eye tracker had detected that one of the three boxes had been selected, the target was presented in one of the two flanking boxes. The authors were interested in which box people would select. To maximise accuracy, people should choose to fixate on the centre box when the boxes were close enough together that the target could still be discriminated in the flanking boxes. When the distance was too large for the target to be visible from the central box, participants should fixate on one or the other side box instead. Surprisingly, it was found that the choice of which box to fixate on did not vary systematically according to the distance between the boxes. The authors concluded that fixation decisions were guided by imperfect heuristics, even in this simplified context in which the best choice of the three fixation locations seemed intuitively obvious. However, is this decision failure specific to eye movements? To answer this question, we adapted their paradigm to study more deliberative, effortful decisions [59]. In one version of the task, we asked participants to choose a place to stand to throw a beanbag into one of two hoops, without knowing which of the two hoops was the target. The logic here was the same as for the detection task: to maximise accuracy, participants should choose to stand at the midpoint between the two hoops when they are close together and next to one hoop or the other when their expected odds of successfully hitting the hoops from the midpoint falls below 50%. Just as in the eye movement task, we found that participants did not modify their choice of standing position with the distance between the hoops. We also observed the same pattern of sub-optimal decisions in a memorization task, where participants were given two strings of digits and did not know which one they would be asked to report. When the digit strings were short, participants should try and memorize both, but when they are too long to remember both accurately, participants should focus on one. Again, we did not find a systematic variation in strategy with the length of the digit string. These tasks demonstrated that the failure to make fixation decisions that optimize accuracy is not unique to the eye movement task, but a more general problem with how we allocate limited resources when faced with multiple goals. This finding aligns with many other instances in the literature in which eye movements have been argued to be a valuable model system for understanding more complex decisions (e.g., [60,61,62,63]).

To explain this failure to optimize task performance in eye movements and other decisions, we suggest that under most natural circumstances, calculating the optimal strategy is not straightforward, as we have already seen from the high computational load associated with executing an optimal series of eye movements to find a Gabor patch in noise [40]. Analogously, in economics, as the number of investment options increases, the advantage of any particular investment strategy shrinks rapidly relative to a simple “1/N” strategy of dividing money equally over N options. In terms of risk-adjusted returns, the 1/N strategy was shown to be virtually indistinguishable from a set of 14 optimal asset allocation strategies for N = 25 [64]. For many decisions, from eye movements to investment strategies, there are more than just two goals to pursue, so a stochastic strategy will be computationally simpler, with outcomes that are indistinguishable from an optimal strategy. We can experimentally create situations where the optimal strategy is simple to calculate and has large benefits, but under most other circumstances, the calculations will be resource-intensive and the benefits far smaller and less predictable. If calculating the expected effects of the full set of potential actions comes at a greater cost under most natural circumstances, perhaps people rely on “habits” as a default to make most of their decisions and do not recognize and adapt their strategy when circumstances arise where better performance is possible.

Given that participants are clearly not recognizing and following optimal strategies in either making eye movements during visual search or in analogous decisions, an interesting question is, what do they do instead? The alternative based on the actions/habits distinction from the animal behaviour literature would be to rely instead on habits. However, “habits” implies rigid repetition of previously-reinforced responses. Defined this way, “habits” do not adequately describe the fixation choices observed in visual search, which are highly variable rather than fixed and rigid. We observed a similar striking range of variability in the choice tasks described above: given exactly the same decision dilemma, most participants exhibited a wide range of different choices from one trial to the next. While participants were consistent in terms of their average behaviour, which was largely insensitive to increasing difficulty, there was surprisingly wide variation around the mean within each individual. To account for this variability, we can again look to historical animal cognition research, where it has been suggested that variability may itself be a reasonable approach to solving problems under conditions of uncertainty [65]. Healthy rats tend to make variable choices when navigating mazes [66] and even seem to prefer mazes that are variable over ones that are fixed [67]. These researchers have suggested that making variable responses allows the animal to explore the “means-end-readiness” of the problem space, to understand the range of possible behaviours and their consequences under uncertain conditions. Bringing this idea back to visual search, within-condition variability is often swept aside as noise in the data, but it may contain important information about how we “solve” search problems, and this could be a useful model for understanding how we solve other problems as well.

### 2.4. Stopping Rules

One key aspect of effective visual search is deciding when the search should be terminated. For example, when searching for missing car keys, when should you stop searching one room and move on to the next? The decision when to stop, both in real life and in a laboratory search task, involves weighing the advantage of saving time (by not searching all possible locations) against missing the target (by not searching for long enough). The decision about when to stop looking can have important effects even in simple, single-fixation search tasks. For example, Dukewich and Klein [68] found shallower search slopes for making present/absent target decisions versus target localization and identification. Rather than being due to differences in the process of finding the target, as one might initially assume, this was likely due instead to the fact that larger sets of distractors cause participants to give up searching before they find the target, leading to reaction times that do not increase with set size as much as they would if people maintained the same level of accuracy across task difficulty. For identification and localization tasks, the reliable presence of a target led to stable error rates over set size. This demonstrates that even in very simple search tasks, it is important to understand the variance in performance that comes from when people decide to give up. The stopping decision will depend largely on people’s knowledge of how likely it is that a target is present, in combination with how much they care about missing it. Circumstances also matter: target prevalence will bias people towards present or absent judgements (rare and extremely rare targets are much more likely to be missed than targets present in half of the trials), but when searching for tumours or weapons, which are rare, but life-or-death, this bias can be over-ridden [20,69]. For search over multiple fixations, there are two levels of stopping decisions. The first is the decision to leave the currently-fixated location and select a new one, which in many respects is an instance of single-fixation search. This level of decision was discussed in detail in a recent paper by Tatler et al. [70], who modelled the process as a threshold based on the relative benefit of the currently fixated target relative to a new fixation. The second level is the decision made after several fixations that it is time to stop looking for a given target altogether. In this discussion, we focus on the second of these.

Early attempts at formalizing stopping rules came from animal foraging models. Charnov [71], in his “marginal value theorem”, postulated that when an animal searches for multiple targets, a single area is searched until the target acquisition rates (so-called “marginal rates”) for this single area fall below the mean number of targets expected to be found in the entire search set. This main premise of the model constitutes its main failure because the forager is expected to know the distribution of the targets in the search array even before he/she searched through the array and because the observer does not differentiate between areas rich in targets and areas containing only a small number of targets. A later model [72] dropped the notions of average acquisition rates per area. Instead, the observer stopped searching an area if the time since the last target was acquired exceeded a certain point. Thus, observers were not expected to know the distribution of the targets across the search arrays prior to search: foragers spent more time searching areas rich in targets and dropped areas with few targets quickly.

Visual search and foraging differ in some fundamental ways: visual search laboratory tasks are normally presented on a computer screen, whereas foraging often involves immersing the participant within the search space. Foraging usually requires more energy, and often visual cues are not available for locating the target [73] (see also [74]). Lastly, foraging for food is more complex than visual search for a target singleton, as it often requires distinction between high- and low-quality patches of food and revisiting the areas that have previously been exhausted [75]. The guided search model addresses the question of when to decide when a single target is absent in a more traditional visual search paradigm, where a single target tends to be present in half the trials [76]. As with the general guided search model described above, the search array is evaluated pre-attentively, and items are selected according to their probability of being a target. Search is terminated when the remaining search items do not reach this probability threshold. This termination threshold is set by the observer to meet a specified speed-accuracy performance trade-off. The main advantage of this model over the earlier models is that the threshold is set flexibly: when error rates are too high, the threshold is lowered to allow longer search times, and when error rates are too low, the threshold is increased, thus increasing RT. Over the course of the experiment, observers acquire implicit knowledge about the duration of successful trials and are more likely to guess as a typical trial duration elapses and the target has not been found.

## 3. Search Strategy and Individual Differences

The majority of the visual search literature has concerned itself with understanding average performance, often expressed in terms of a set-size × reaction time slope. For many aspects of search, this is an entirely appropriate approach to hypothesis testing, but for other aspects, it is less appropriate. A recent paper by Hedge and colleagues [77] made the important point that “reliability” has two very distinct meanings in experimental psychology: on the one hand, it can mean how consistent a given effect size is across different samples, and on the other, it can mean how reliably the same sample produces the same results. The first definition of reliability is used in the context of interpreting averaged data and is important for ensuring a particular experimental manipulation is reproducible. The second definition of reliability depends on individual performance being stable over repeated measurements, which is important for correlational studies that attempt to understand variation in a particular effect size. Hedge and colleagues noted that not only are these two types of reliability very distinct conceptually, they are also to some extent mutually exclusive: low variation between individuals makes averages more reliable, but restricts the range for detecting correlations with other variables. Conversely, the large and stable differences between individuals that make correlations reliable only contribute noise to analyses of averaged data. In the current context, this is a particularly important distinction, because visual search involves a complicated set of sub-processes, some of which may be best studied using an averaging approach, and some of which need to be understood by examining individuals as the basic unit of analysis.

**Figure 2:**
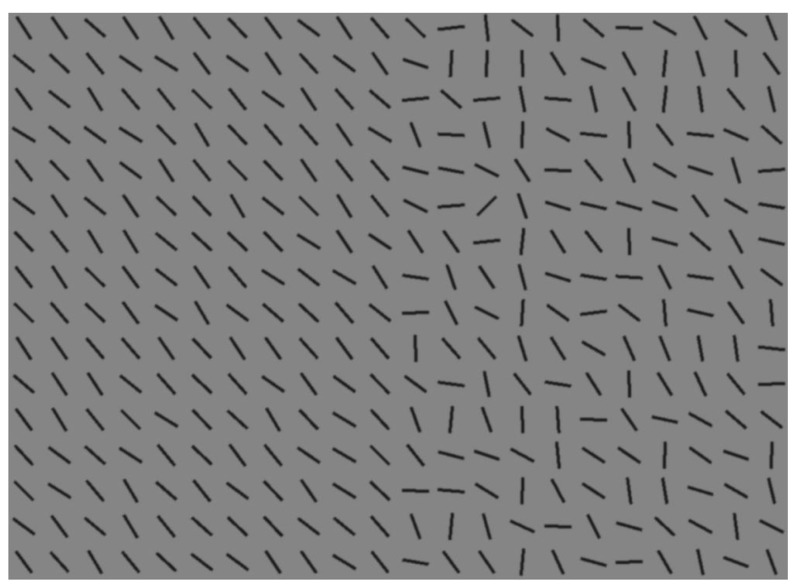
Example stimulus from [78]. The target is the line segment that is perpendicular to the mean orientation of the distractors. In this example, it is nine items from the right, six down.

**Figure 3:**
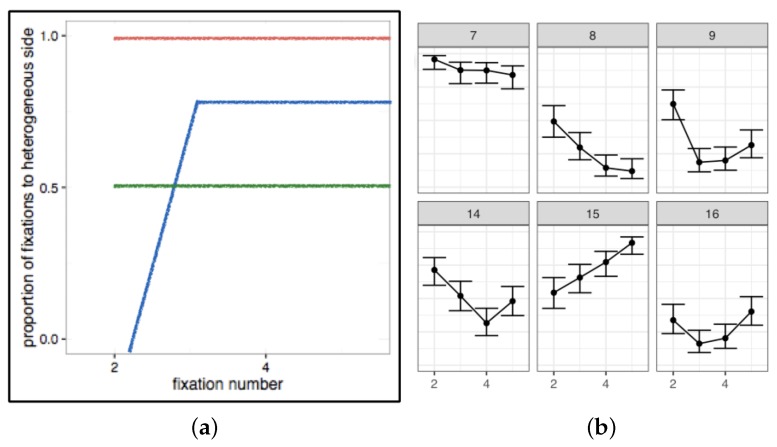
(**a**) The red and blue lines show how an optimal and close-to-optimal observer should search the split-half stimuli. The green line shows what we would expect from a stochastic searcher. (**b**) Data from six participants [78]. We can see that while Participant 7 approached the optimal strategy, and 15 could be considered close-to-optimal, other participants (14, 16) behaved in line with the predictions from the stochastic search strategy. Furthermore, Participants 8 and 9 appeared to be implementing a “counter-optimal” strategy.

Data averaged across individuals not only hides important information, it can also sometimes lead to spurious conclusions. An example of this can be seen in our recent work on visual search strategies [78]. Participants searched an array of randomly-orientated line segments, as shown in Figure 2. The target was visible using peripheral vision on the homogeneous side of the array; eye movements to this side of the array provided no new information. We created these stimuli to distinguish between optimal (in which the searcher should fixate only on the heterogeneous half of the array) and stochastic strategies (in which both halves should be fixated equally often). If we use the average performance over our participants and compare to our hypotheses (Figure 3a), then it looks like the data support the stochastic search model. However, such a conclusion would be wrong, as when we examine how each individual approached this visual search task, we found large, and stable, differences, as shown in Figure 3b: about a third of our participants approached ideal performance, while another third did the opposite, leading to performance that was worse than if a purely random strategy had been followed (the remaining third was somewhere in-between).

A similarly striking range of individual differences in the search task was reported by Irons and Leber ([79], see also [80]) with their adaptive choice visual search task (ACVS) [79]. In their paradigm, participants had to search for a numeral 1–5 in a small red or blue square. On each trial, there was both a red and blue target, embedded within a collection of red, blue, green, and variable distractors. The variable coloured distractors started out red (in which case the optimal strategy was to search for a blue target) and, upon each successive trial, slowly turned from red to purple to blue. For trials in which there were more blue distractors than red, participants should search for the red target, and vice versa. Whereas group performance was far from optimal, some observers switched to the easier target as the distractors changed colour (although they still failed to switch at an optimal point); some always searched for the same colour and avoided switching; and some frequently switched between targets, but not in a way that related to the distractor colours. The flexible adjustment of attention to colour and the differences between individuals in this ability highlight the limitations of salience and guidance alone in accounting for visual search performance.

In another study, observers had to find multiple targets while ignoring distractors in a search array. The targets were defined by either one (e.g., colour) or two features (e.g., colour and shape) [81,82]. When the target was defined by a single feature, observers frequently switched between target types (e.g., picking a yellow target, then red, then yellow again). When observers searched for a target defined by two features (shape and colour), most of them tended to pick the same target type in long runs (e.g., all yellow squares) until they their exhausted search and then moved on to search for the other type of the target (e.g., red triangles). Notably, four out of 16 participants, termed “super-foragers”, continued to use the same strategy in the two conditions, but with no apparent cost to performance. In other words, a minority of people seem able to hold two target templates in mind simultaneously. In an earlier demonstration of individual differences in search strategy, Araujo and colleagues found that the majority of participants chose inefficient search strategies when planning a saccade to one of the two possible target locations [83]. In this experiment, the target was a randomly-oriented letter T located in one of two clusters of letter Ls. The probability of the target occurring in one of the clusters was 80% and was signalled to the observers by the different luminance levels of the two clusters. The distance between each cluster of letters and central fixation varied between the trials. Because the display was presented for only 500 msc, the best performance would be achieved by inspecting the high probability location first. Yet, the cue signalling the higher probability was only used by one out of the six participants. The other five observers preferred to inspect the closer cluster first (to varying degrees) and then the remaining cluster, even though the display had been removed before the second saccade arrived.

One might be tempted to conclude that some people are simply better at search than others, but our recent work suggested that something more complex underlies these differences [84]. We tested the same set of 64 participants with the split-half [78], adaptive choice [79] and foraging [82] paradigms. Although each of these tasks has been shown to have a good test-retest reliability (r≈ 0.7–0.9), we found a surprising lack of correlations across tasks. Not only was there a wide range of differences in how people approached a visual search task, but these individual differences depended critically on task structure. Given the range of performance we observed in these three tasks, it is possible that the interaction between these two factors (individual differences and task structure) was responsible for far more of the variation we see in human search behaviour than has been explained by visual salience and guided search. The important question that remains to be understood is not whether people are optimal searchers or not, but rather why some people are optimal and under what circumstances. Given that this person–circumstance interaction accounted for the majority of variance in these three different search tasks, understanding it will not only help us build more powerful models of visual search itself, but it also has the potential to facilitate efficient search in industrial and social circumstances (two particularly high-stakes examples are security and health care).

Individual differences in search can also have importance in patient populations. We observed a wide range of individual differences in a series of experiments looking at how eye movement strategies of healthy people are affected by simulated visual deficits [85]. Visual information in one hemifield was removed on-line while participants searched a display of lines for the target (line tilted $45^{\circ }$ to the right) embedded in an array of homogeneously- or heterogeneously-oriented lines, similar to those shown in Figure 2, except the arrays were not split into two halves; instead, distractor orientations were sampled from the same range across the array (either homogeneous or heterogeneous). Making eye movements to the initially-sighted side did not harm performance for a heterogeneous background, but for the homogeneous background, the logic was similar as for the split/half arrays described above: a target on the visible half of the display could already be detected, without the need for any eye movements. The effective strategy was therefore to make large eye movements to the blind side, to reveal the part of the display that was currently hidden. We found that participants continued to use the inefficient strategy of making eye movement to the sighted side even when the search was very easy and the target could be easily ascertained to not be present in the periphery. When we exposed participants to the simulated visual deficits over five days, with financial incentives for performance improvements, the majority of participants became gradually more efficient [86], but those who were inefficient at the beginning of the experiment were still far from optimal, while individual differences in strategy were very stable over a week of repeated exposure to the task. Variability in performance between patients with visual field deficits has often been attributed to factors such as the site and extent of the lesion and the age at the onset (see for example [87,88]). One gap in patient studies that our research on individual differences highlighted is the knowledge of premorbid strategies. We cannot reasonably assume that the patients will show optimal performance post-lesion if their premorbid strategy was poor, and we have shown that these differences are large and persistent in healthy populations.

Variation in abilities has been extensively explored elsewhere, for instance in the process of comparing shapes [89] and in the face recognition literature, where it has been shown that observers vary on a spectrum from developmental prosopagnosia (profound inability to recognise familiar faces) to super-recognisers (observers that are significantly better than average at recognising faces [90]). Studies of twins suggest that a genetic component might drive the differences, and the ability to recognise faces is independent of more general aspects of cognition such as attention and intelligence [91]. Much can be learned about a particular skill by understanding the full range of individual differences, and visual search research, like many other areas of psychology, has not yet realized the full potential of this approach.

## 4. Conclusions

Visual search continues to be a useful model task and a rich source of data for understanding a wide range of perceptual, cognitive, and motor skills. Much has already been learned about perception and attention from studying eye movements and attention during visual search tasks. Much remains to be understood about strategy, bias, and decision, and visual search is poised to provide insights into these domains, as well. In our conclusions, therefore, we offer four inter-related recommendations for future research into visual search to tackle, to move the field toward a more complete understanding of this complex, but important task.

1. The focus of our experiments and analyses should not only be to explain average patterns, but also to account for variance. The large sources of variance, relative to smaller ones, will be the more powerful predictors of search performance. If we can understand and control these, the smaller sources of variance will be easier to tackle. Individual differences and variability within conditions should not be hidden away in averaged data, but made a central part of our models and theories.
2. A related suggestion is to be cautious in interpreting measures of central tendency, such as means and medians. Given the large range of individual differences we have observed in most of our own search data, and the spurious conclusions we could have reached if we relied on average patterns alone, we think it is important to consider carefully whether a measure of central tendency is, in fact, a good representation of a particular set of data. That is, is the mean (or median) pattern similar to most of the trial and individual level results? If a particular summary statistic is not an accurate or adequate representation of most of your data, do not report it. Instead, show the full range of results so other researchers can understand how variable a given behaviour is within and between individuals. This variance does not indicate a failed manipulation or “noisy data”; instead, consider that it contains essential information, without which we cannot fully understand visual search performance.
3. Based on observations of independent sources of variance across different tasks [84,89], it is clearly important to address directly the question of how confidently we can apply conclusions from one search task to related and unrelated tasks and contexts. For example, we often assume that visual primitives like line segments and Gabor patches will scale up to more complex scenes and objects or that basic phenomena like attentional capture or inhibition of return will be easy to observe in real-world situations. In fact, it is difficult to find straightforward instances in the literature where these basic effects have clearly generalized from the laboratory to more complex real-world situations. It is important to note that the context-specificity of a given effect is not an indication that it is trivial or unimportant. Instead, it is an important source of data for understanding the constraints and boundary conditions for patterns of results that can be reliably produced in the laboratory. Directly measuring how particular manipulations and interventions affect search in a variety of situations can be a fruitful source of insight into these effects.
4. We all have a tendency to stay within the bounds of familiar theories and models. Looking outside the vision and attention literature can lead to many new useful ideas and explanations, especially in visual search, which is a rich and complex task. Our understanding can be enriched from insights and models from other fields such as decision-making, learning, human factors and individual differences.
